# Real-world clinical outcomes and treatment patterns in patients with MDD treated with vortioxetine: a retrospective study

**DOI:** 10.1186/s12888-023-05439-8

**Published:** 2023-12-13

**Authors:** Brandon T. McDaniel, Victor Cornet, Jeanne Carroll, Lambros Chrones, Joseph Chudzik, Jeanette Cochran, Shion Guha, Debra F. Lawrence, Maggie McCue, Sara Sarkey, Betty Lorenz, Jay Fawver

**Affiliations:** 1 Parkview Mirro Center for Research and Innovation, 10622 Parkview Plaza Drive, Fort Wayne, IN 46845 US; 2 Takeda Pharmaceuticals U.S.A., Inc, Lexington, MA US; 3 Faculty of Information, Department of Computer Science, University of Toronto, Toronto, ON Canada; 4 Parkview Physicians Group − Mind-Body Medicine, Fort Wayne, IN US

**Keywords:** Depression severity, Major depressive disorder, Patient-reported outcome measures, Real-world clinical practice setting, Vortioxetine, Comorbid psychiatric diagnoses, Previous antidepressant treatments, Sexual dysfunction, Cognitive functioning, Social functioning

## Abstract

**Background:**

This study included evaluation of the effectiveness of vortioxetine, a treatment for adults with major depressive disorder (MDD), using patient-reported outcome measures (PROMs) in a real-world setting.

**Methods:**

This retrospective chart review analyzed the care experiences of adult patients with a diagnosis of MDD from Parkview Physicians Group – Mind-Body Medicine, Midwestern United States. Patients with a prescription for vortioxetine, an initial baseline visit, and ≥ 2 follow-up visits within 16 weeks from September 2014 to December 2018 were included. The primary outcome measure was effectiveness of vortioxetine on depression severity as assessed by change in Patient Health Questionnaire-9 (PHQ-9) scores ~ 12 weeks after initiation of vortioxetine. Secondary outcomes included changes in depression-related symptoms (i.e., sexual dysfunction, sleep disturbance, cognitive function, work/social function), clinical characteristics, response, remission, and medication persistence. Clinical narrative notes were also analyzed to examine sleep disturbance, sexual dysfunction, appetite, absenteeism, and presenteeism. All outcomes were examined at index (start of vortioxetine) and at ~ 12 weeks, and mean differences were analyzed using pairwise *t* tests.

**Results:**

A total of 1242 patients with MDD met inclusion criteria, and 63.9% of these patients had ≥ 3 psychiatric diagnoses and 65.9% were taking ≥ 3 medications. PHQ-9 mean scores decreased significantly from baseline to week 12 (14.15 ± 5.8 to 9.62 ± 6.03, respectively; *p* < 0.001). At week 12, the response and remission rates in all patients were 31.0% and 23.1%, respectively, and 67% continued vortioxetine treatment. Overall, results also showed significant improvements by week 12 in anxiety (*p* < 0.001), sexual dysfunction (*p* < 0.01), sleep disturbance (*p* < 0.01), cognitive function (*p* < 0.001), work/social functioning (*p* = 0.021), and appetite (*p* < 0.001). A significant decrease in presenteeism was observed at week 12 (*p* < 0.001); however, no significant change was observed in absenteeism (*p =* 0.466).

**Conclusions:**

Using PROMs, our study results suggest that adults with MDD prescribed vortioxetine showed improvement in depressive symptoms in the context of a real-world clinical practice setting. These patients had multiple comorbid psychiatric and physical diagnoses and multiple previous antidepressant treatments had failed.

**Supplementary Information:**

The online version contains supplementary material available at 10.1186/s12888-023-05439-8.

## Introduction

Major depressive disorder (MDD) remains a serious health problem in the United States (US), with a 12-month prevalence of more than 10% in the adult population [1]. During the recent COVID-19 pandemic, prevalence of symptoms of anxiety disorder and depressive disorder increased more than 3-fold in the US [2, 3]. Numerous studies have shown that a high proportion of patients with MDD experience modest rates of response and remission after antidepressant treatment [4, 5], and a lack of remission increases risk of relapse and recurrence and decreases quality of life [5].

Vortioxetine is a multimodal antidepressant approved for the treatment of MDD in adults [6, 7]. It works through inhibition of the 5-HT (serotonin) transporter, as well as direct effects on multiple 5-HT receptors (5-HT_3_, 5-HT_7_, and 5-HT_1D_ receptor antagonist; 5-HT_1B_ partial agonist; 5-HT_1A_ agonist) [7]. Vortioxetine has an established safety and tolerability profile and has demonstrated efficacy in multiple clinical studies over the dose range of 5 to 20 mg/day, reducing depression symptom severity and improving certain aspects of cognitive and sexual dysfunction associated with prior treatment with certain serotonergic agents [6]. Additionally, in published placebo-controlled trials and open‐label extension studies, vortioxetine demonstrated a lower incidence of secondary effects, such as insomnia-related events and weight gain [7]. However, there are few studies of vortioxetine in the real world, especially studies using patient-reported outcome measures (PROMs) [8].

Differences are often found between clinical trial outcomes and those observed during mental health treatment in more naturalistic settings. One study found that although two-thirds of patients receiving psychotherapy demonstrated improvement in clinical trials within 13–18 sessions, fewer than 25% of patients achieved meaningful improvement and received only 3–5 sessions when treated with psychotherapy under real-world conditions [9]. The study also noted that, in real-world settings, patients rarely underwent 18 sessions of therapy. Yet, systematic monitoring of the presence and severity of depressive symptoms and response to treatment, allowing for adjustments to treatment, has been shown to be important for improving outcomes in MDD [5]. Thus, real world studies are needed to fill the gap between clinical research and routine clinical practice.

One of the main contributors to the poorer observed outcomes in mental healthcare is a lack of systematic follow-up to detect patients who are not responding to treatment [10]. Only about one-third of patients who experience increased symptom severity are detected through routine clinical judgment by their therapists [11]. Yet, the use of symptom rating scales to monitor outcomes can help change the treatment plan when patients are not responding to treatment [12]. Measurement-based care (MBC) is a systematic approach to evaluating clinical outcomes and using the results to guide and inform care plans and treatment decisions [13]. MBC involves a step-by-step approach to delivering clinical care through routine assessments, such as measuring the severity of symptoms with rating scales, treating and reviewing outcomes, and using these assessments in decision-making to alter the patient care plan as needed based on data collected throughout treatment [14, 15]. Although numerous validated symptom rating scales exist to measure changes in symptom severity over time, they are underused; fewer than 20% of healthcare professionals routinely administer them to their patients with depression/anxiety [11, 16]. Despite infrequent use, MBC has been shown to improve clinical outcomes and quality of care [10, 17].

Several clinician-rated outcome measures and PROMs can be considered for MBC. The National Committee for Quality Assurance (NCQA) has proposed depression symptom monitoring with the Patient Health Questionnaire-9 (PHQ-9), a PROM, for the Healthcare Effectiveness Data and Information Set (HEDIS), one of healthcare’s most widely used performance improvement tools [18]. PROMs provide direct feedback on patients’ progress with a particular treatment, such as improvement in core depressive symptoms, functioning, quality of life, and work productivity, thereby allowing healthcare practitioners to adjust treatment as needed [5, 19]. While clinician-rated scales tend to be more comprehensive, PROMs generally take less time to complete, and have been shown to be equivalent in their ability to identify treatment responders and remitters [20, 21]. The use of PROMs to monitor outcomes in the treatment of depression is now recommended by several medical societies and organizations in the US, United Kingdom, and Europe, including the US Health Resources and Services Administration, American Psychiatric Association, American Psychological Association, UK National Institute for Health and Care Excellence, NHS England Increasing Access to Psychological Therapies program, and the Nederlands Huisartsen Genootschap (Dutch Society of General Practitioners) [19]. The PHQ-9 is one of the most often used depression screening tools in adults and has demonstrated clinical utility and diagnostic accuracy [22]. It is a convenient and effective screening tool used to monitor treatment effect and severity of depression, and may help improve the management of MDD. Evidence from previous studies indicates that combining screening with systems of care improves outcomes and long-term remission in depression care [23, 24]. Similarly, the Generalized Anxiety Disorder-7 (GAD-7) questionnaire is a patient-rated, 7-item measure of anxiety symptoms that has been shown to be a quick and efficient tool for screening for GAD and has been validated in multiple studies [25].

In addition to primary symptoms of depression, patients with MDD also exhibit impairment in one or more areas of functioning, sleep, and/or sexual dysfunction. Patients’ perspectives can provide important insights into other depression-associated symptoms for which they seek relief. The Perceived Deficits Questionnaire-20 (PDQ-20) is one of the more extensively validated patient-reported instruments for the assessment of subjective cognitive dysfunction in patients with depression [26]. The Work and Social Adjustment Scale (WSAS) is a short, quick, validated, self-reported tool directly developed for the assessment of workability and social functioning in patients with mental health problems [27, 28]. The Patient-Rated Inventory of Side Effects (PRISE) is a 7-item scale that measures the presence of side effects in 8 organ system domains, as well as other side effects [29].

Another measure that captures patient perspectives is the clinician narrative notes. Unstructured narratives in the clinical notes can offer key details about patients’ signs and symptoms, especially when notes are documented in a standardized way, and can be converted into powerful insights [30]. Many clinical notes follow a traditional subjective, objective, assessment, plan (SOAP) approach that may include subjective statements about relevant patient behavior or mood with measurable and quantifiable observable data, along with the physician’s assessment and recommendation for a treatment plan [31]. Recently, several other formats that include a combination of a template and free text have evolved; these keep a structured format while allowing anyone involved in the patient’s care team to easily extract pertinent information [31]. For instance, at the Parkview clinical practice site, standardized progress note templates provide consistency in charting for all providers so that chart review can be efficiently completed.

The objectives of our study included evaluation of the care experiences of patients with MDD and the effectiveness of vortioxetine on patient outcomes from baseline to 12 weeks as determined using PROMs and clinical narrative notes. These included examining scores related to depression (PHQ-9), anxiety (GAD-7), sleep disturbance (PRISE and clinical notes), sexual dysfunction (PRISE and clinical notes), appetite (clinical notes), absenteeism (clinical notes), weight/body mass index (BMI), cognitive functioning (PDQ-20), and work and social functioning (WSAS).

## Methods

### Study design and patient population

A retrospective chart review was conducted of the care experiences of patients with MDD from Parkview Physicians Group − Mind-Body Medicine, an outpatient psychiatric practice in the Midwestern US. Specifically, we reviewed the charts of patients aged ≥ 18 years with a diagnosis of MDD, a prescription for vortioxetine,^1^ an initial visit, and at least 2 follow-up visits within 16 weeks after baseline (all within the time frame of September 1, 2014, to December 31, 2018). Patients with a diagnosis of bipolar depression and/or schizophrenia were excluded. As the current study was a retrospective chart review without any interaction with human participants, the study was determined by the Parkview Institutional Review Board (IRB) to qualify as being exempt from IRB oversight [per the criteria in 45 CFR 46,104(d)(4)]. We extracted the following information from the charts: historical and concurrent diagnoses, medication history, age, race/ethnicity, vortioxetine dosage, and scores on patient-reported outcome measures (PHQ-9, GAD-7, PDQ-20, PRISE, and WSAS). We also extracted the clinical narrative notes.

### Outcome measures

The primary outcome measure was effectiveness of vortioxetine in treating depression severity (assessed by change from baseline in PHQ-9 score at 12 weeks after initiation of vortioxetine). The PHQ-9 [32] is a patient-reported outcome for screening, diagnosing, monitoring, and measuring the severity of depression. Global scores ranged from 0 (absence of depression) to 27 (severe depression); remission is defined as a global score < 5 [33].

Secondary outcomes included changes in anxiety symptoms and cognitive symptoms, improvement in sexual dysfunction and sleep disturbance, and change in work and social functioning. Changes in anxiety symptoms were assessed by GAD-7 [34], a patient-reported 7-item scale with a global score ranging from 0 (absence of anxiety) to 21 (severe anxiety). Scores of 5, 10, and 15 represent cut-points for mild, moderate, and severe anxiety, respectively.

Changes in cognitive symptoms were assessed by PDQ-20 [35, 36], a 20-item questionnaire that generates a total score and 4 subscale scores (attention/concentration, retrospective memory, prospective memory, and planning/organization); this was originally used in patients with multiple sclerosis but has been adapted and validated for use in psychiatry. The total score was utilized in the current study.

Improvement in sexual dysfunction and sleep disturbance was assessed by PRISE [29], a 7-item patient-reported outcome in which the patient rates the symptoms as tolerable or distressing for the following symptom domains: gastrointestinal, heart, skin, nervous system, eyes/ears, genital/urinary systems, sleep, sexual functioning, and other.

Change in work and social functioning was assessed with WSAS [28], a patient-reported outcome with a 5-item scale in which each item is rated on a 9-point scale ranging from 0 (not at all a problem) to 8 (very severely impaired). The total score ranges from 0 to 40, with a high score indicating greater dysfunction.

Analysis of clinical narrative notes was performed using an algorithm developed in MATLAB® Professional Edition R2020a by the study team at the Parkview Mirro Center for Research and Innovation. First, clinical notes for at least 500 patients were manually reviewed to determine categories and key words related to sleep, sexual dysfunction, appetite, presenteeism, and absenteeism. Scoring spreadsheets (one per domain) were then compiled, giving our algorithm instructions on how key words should be used to code encounter notes by using regular expressions. Scores were validated by comparing the manual scores with the scores returned by the algorithm for a random 50 clinical notes to calculate sensitivity, specificity, precision, and accuracy. Overall, the algorithm provided satisfactory results for most categories, with sensitivity and specificity greater than 80% and accuracy greater than 90% on the validation dataset (see Additional file 1 - Additional Table 1, Validation Results for the Overall Scores by Domain, and Additional Table 2, Validation Results for Individual Category Scores Under Various Domains). The algorithm was then applied on all clinical notes to get baseline and 12-week scores for each patient.

Other descriptive variables and secondary outcomes included demographic and clinical characteristics (dosage at baseline and week 12, psychiatric history, other diagnoses, and medication history), weight/BMI change, response, remission, and persistence rates. Response was defined as proportion of patients with at least a 50% reduction in their PHQ-9 score from baseline to 12 weeks. Remission was defined as the percentage of patients with PHQ-9 score ≤ 4 by 12 weeks. Persistence rates were assessed as the percentage of patients who continued to use vortioxetine at 12 weeks. Vortioxetine dosage was examined at the index date and 12 weeks, and mode and median dose and percentages of patients at 5 mg, 10 mg, or 20 mg at index and 12 weeks were reported. Historical and concurrent psychiatric and other diagnoses were examined prior to and during vortioxetine use, and patients who had each diagnosis, mean number of these diagnoses, and percentage of patients who had 1, 2, or 3 or more were reported. Medications taken by the patients prior to and concurrently with vortioxetine use were examined, and percentage and mean number of each medication and percentage of patients who had 1, 2, or 3 or more diagnoses were reported.

### Data analysis

All patients included in the study were required to have at least 3 time points of data, including baseline (start of vortioxetine) and at least 2 follow-up visits within 16 weeks of starting vortioxetine. The index date (baseline) for each patient was defined as the first day of initiation of vortioxetine. The 12-week time point was defined as the encounter closest to 12 weeks and within a window of 8 to 16 weeks (mean = 83.67 days since index; SD = 14.06). Descriptive statistics were used with continuous variables represented as mean values (± SD) or median values (ranges), and categorical variables reported as numbers and percentages. Mean differences between the index date and 12 weeks were analyzed using pairwise *t* tests. The effect size for a paired-samples *t* test was determined by Cohen’s *d* [37], which was calculated by dividing the mean difference by the standard deviation of the difference, as shown below:

Cohen’s *d* = mean_D_ ⁄SD_D_, where D is the difference of the paired samples values.

For example, a Cohen’s *d* of 0.5 indicates that the two group means differ by 0.5 SD, and a Cohen’s *d* of 1 indicates that the group means differ by 1 SD. A Cohen’s *d* of 0.2 is considered a “small” effect size, 0.5 represents a “medium” effect size, and 0.8 a “large” effect size.

## Results

### Patient characteristics and demographics

A total of 1242 patients diagnosed with MDD were included in the analysis. Baseline patient demographics are shown in Table 1. Patients had a mean ± SD age of about 46 ± 17 years, and 91% were White. Women (68%) accounted for a majority of the patient population (Table 1). In the patient sample analyzed, median dose of vortioxetine was 5 mg at index and 10 mg at 12 weeks. In addition, at 12 weeks, 17% of patients were taking 5 mg daily, 53% were taking 10 mg daily, and 30% of patients were taking 20 mg daily.

**Table 1:** Baseline characteristics and demographics of patients

Characteristics	*N*	Mean ± SD (range)
Age, years	1242	45.9 ± 16.5 (18–90)
Height, inches	1238	66.7 ± 4.1 (57–80)
Weight, lb	1208	192.0 ± 51.7 (88.6–492.6)
BMI, kg/m^2^	1207	30.3 ± 7.5 (15.2–69.7)
**Sex**	***N***	**%**
Female	838	67.5%
Male	404	32.5%
**Race**	***N***	**%**
White	1131	91.1%
Patient declined	67	5.4%
Black or African American	22	1.8%
Hispanic or Latino	10	0.8%
Asian	5	0.4%
Unknown	4	0.3%
American Indian or Alaska Native	2	0.2%
Native Hawaiian or Pacific Islander	1	0.1%

*Note*: BMI = body mass index

### Patient medical history

Overall, 64% of patients had ≥ 3 psychiatric diagnoses during their vortioxetine treatment (Table 2). The 3 most frequent psychiatric diagnoses were generalized anxiety disorder (GAD) (83%), moderate recurrent MDD (68%), and MDD single episode (26%) during vortioxetine treatment (Table 2). Attention-deficit hyperactivity disorder (26%), social phobia (26%), and posttraumatic stress disorder (24%) were the next most common psychiatric disorders. Eating disorders, obsessive-compulsive disorder, panic disorder, and disorders of adult personality and behavior were seen in fewer than 10% of patients. Alcohol-related disorders and other psychoactive substance-related disorders were relatively uncommon, with most occurring in fewer than 5% of patients.

**Table 2:** Historical and concurrent psychiatric diagnoses

Psychiatric diagnoses	Concurrent n (%)	Historical n (%)
**Number of psychiatric diagnoses per patient, ** ***mean (SD)***	*3.09 (1.29)*	*3.14 (1.34)*
**Number of psychiatric diagnoses per patient**		
1 diagnosis	75 (6.0)	79 (6.4)
2 diagnoses	373 (30.0)	362 (29.1)
3 diagnoses or more	794 (63.9)	801 (64.5)
Generalized anxiety disorder	1029 (82.9)	1013 (81.6)
Major depressive disorder, recurrent, moderate	842 (67.8)	837 (67.4)
Major depressive disorder, single episode	326 (26.2)	390 (31.4)
Attention-deficit hyperactivity disorder	325 (26.2)	321 (25.8)
Social phobia	321 (25.8)	324 (26.1)
Posttraumatic stress disorder	293 (23.6)	291 (23.4)
Major depressive disorder, recurrent, in remission, unspecified	192 (15.5)	178 (14.3)
Major depressive disorder, recurrent, mild	162 (13.0)	159 (12.8)
Major depressive disorder, recurrent, in partial remission	131 (10.5)	106 (8.5)
Eating disorders	91 (7.3)	93 (7.5)
Major depressive disorder, recurrent severe without psychotic features	77 (6.2)	85 (6.8)
Major depressive disorder, recurrent, in full remission	76 (6.1)	81 (6.5)
Cannabis-related disorders	75 (6.0)	77 (6.2)
Disorders of adult personality and behavior	72 (5.8)	69 (5.6)
Obsessive-compulsive disorder	71 (5.7)	66 (5.3)
Major depressive disorder, single episode, moderate	70 (5.6)	74 (6.0)
Panic disorder	67 (5.4)	77 (6.2)
Dysthymic disorder	55 (4.4)	58 (4.7)
Alcohol-related disorders	51 (4.1)	58 (4.7)
Nicotine dependence	43 (3.5)	66 (5.3)
Major depressive disorder, recurrent	40 (3.2)	42 (3.4)
Major depressive disorder, recurrent, unspecified	39 (3.1)	44 (3.5)
Other anxiety disorders	36 (2.9)	43 (3.5)
Major depressive disorder, single episode, mild	31 (2.5)	38 (3.1)
Opioid-related disorders	24 (1.9)	30 (2.4)
Major depressive disorder, single episode, in full remission	17 (1.4)	21 (1.7)
Alzheimer’s dementia	17 (1.4)	18 (1.4)
Major depressive disorder, single episode, severe without psychotic features	12 (1.0)	15 (1.2)
Other stimulant-related disorders	10 (0.8)	9 (0.7)
Tic disorder	10 (0.8)	9 (0.7)
Major depressive disorder, single episode, in partial remission	8 (0.6)	12 (1.0)
Other psychoactive substance–related disorders	7 (0.6)	15 (1.2)
Major depressive disorder, single episode, severe with psychotic features	6 (0.5)	7 (0.6)
Premenstrual dysphoric disorder	5 (0.4)	6 (0.5)
Major depressive disorder, recurrent, severe with psychotic symptoms	5 (0.4)	8 (0.6)
Cocaine-related disorders	5 (0.4)	5 (0.4)
Sedative-, hypnotic-, or anxiolytic-related disorders	4 (0.3)	6 (0.5)
Dissociative and conversion disorders	4 (0.3)	6 (0.5)
Impulse disorders	4 (0.3)	4 (0.3)
Vascular dementia	2 (0.2)	4 (0.3)
Dementia in other diseases classified elsewhere	0 (0)	0 (0)
Frontotemporal lobe dementia	0 (0)	0 (0)
Dementia with Lewy bodies	0 (0)	0 (0)
Hallucinogen-related disorders	0 (0)	0 (0)
Inhalant-use disorder	0 (0)	0 (0)

On average, patients had at least 2 physical diagnoses, with 18% having no concurrent diagnoses other than psychiatric disorders, 25% having 3 or more comorbidities, and 48% having ≥ 2 comorbidities (Table 3). The most frequent comorbidities were psychophysiological insomnia (48%), obesity (BMI ≥ 30; 45%), and sleep disorders (37%) (Table 3). Migraine (19%), hypertension (17%), and hypothyroidism (14%) were the next 3 most common comorbidities. Type 2 diabetes, chronic ischemic heart disease, hyperthyroidism, and cancer were less common, occurring in fewer than 5% of patients. A total of 78% and 57% of patients were receiving 3 or more drug class-based regimens prior to and at the index date, respectively (Table 4). Prior to the index date, treatment with an antidepressant and/or related medication class was common. About 60% of patients had a prescription for selective serotonin reuptake inhibitors (SSRIs), 43% were using serotonin norepinephrine-reuptake inhibitors (SNRIs), and 45% were using norepinephrine and dopamine reuptake inhibitors (NDRIs) and tetracyclics (Table 4). Antihyperlipidemics (33%), antihypertensives (28%), and antipsychotics (24%) were also commonly used prior to the index date. Additional medications such as beta blockers (21%), tricyclic agents (15%), diuretics (16%), hematological agents (14%), antidiabetics (12%), and calcium blockers (11%) were also used before the index date. A total of 66% were taking ≥ 3 other medications prescribed for MDD and comorbidities while receiving vortioxetine treatment (Table 4). Also, while patients were receiving vortioxetine treatment, other serotonin modulators (39%) were the most common treatment class used, followed by SNRIs (35%), NDRIs and tetracyclics (34%), SSRIs (30%), antihyperlipidemics (29%), antihypertensives (28%), antipsychotics (26%), nonbenzodiazepine hypnotics (22%), and benzodiazepines (20%).

**Table 3:** Historical and concurrent other diagnoses/comorbidities

Physical diagnoses	Concurrent	Historical
Number of other diagnoses per patient, mean (SD)	1.72 (1.36)	1.81 (1.55)
**Number of other diagnoses per patient, ** ***N*** ** (%)**		
None	220 (17.7)	254 (20.5)
1 diagnosis	430 (34.6)	377 (30.4)
2 diagnoses	286 (23.0)	277 (22.3)
3 diagnoses	306 (24.6)	334 (26.9)
Psychophysiological insomnia^a^	590 (47.5)	464 (37.4)
Obesity^b^	564 (45.4)	555 (44.7)
Sleep disorders	465 (37.4)	521 (41.9)
Migraine	235 (18.9)	238 (19.2)
Hypertension	205 (16.5)	251 (20.2)
Hypothyroidism	173 (13.9)	194 (15.6)
Restless leg syndrome	72 (5.8)	65 (5.2)
Type 2 diabetes	62 (5.0)	75 (6.0)
Fibromyalgia	59 (4.8)	65 (5.2)
Chronic pain	38 (3.1)	51 (4.1)
Chronic ischemic heart disease	35 (2.8)	44 (3.5)
Cancer	31 (2.5)	30 (2.4)
Sleep apnea, unspecified	31 (2.5)	44 (3.5)
Constipation	22 (1.8)	50 (4)
Primary insomnia	15 (1.2)	39 (3.1)
Cerebral infarction (stroke)	9 (0.7)	18 (1.4)
Type 1 diabetes	7 (0.6)	11 (0.9)
Sleep disorders not due to a substance or known physiologic condition	7 (0.6)	8 (0.6)
Hypoactive sexual desire disorder	3 (0.2)	3 (0.2)
Sexual disorders	2 (0.2)	2 (0.2)
History of myocardial infarction	1 (0.1)	2 (0.2)
Hyperthyroidism	1 (0.1)	4 (0.3)
Male erectile disorder	1 (0.1)	1 (0.1)
Primary hypersomnia	0 (0)	0 (0)
Female sexual arousal disorder	0 (0)	0 (0)
Female orgasmic disorder	0 (0)	0 (0)
Male orgasmic disorder	0 (0)	1 (0.1)
Sexual aversion disorder	0 (0)	0 (0)
Obstructive sleep apnea (adult) (pediatric)	0 (0)	0 (0)

^a^Psychophysiological insomnia is an ICD-10 diagnosis

^b^Obesity defined as having a diagnosis of obesity in their health record or having a body mass index ≥ 30

ICD-10, *International Statistical Classification of Diseases, Tenth Revision*

**Table 4:** Historical and concurrent other medications/drug classes prescribed for MDD and comorbidities

		Concurrent	At index	Historical
Number of other medications/drug classes, mean (SD)		3.71 (2.2)	3.23 (2.1)	4.81 (2.9)
**Number of medications/drug classes per patient, ** ***N*** ** (%)**				
None		45 (3.6)	69 (5.6)	34 (2.7)
1		153 (12.3)	187 (15.1)	87 (7.0)
2		222 (17.8)	279 (22.5)	159 (12.8)
3 or more		822 (65.9)	707 (56.9)	962 (77.5)
**Drug class**	**Drug**	***N*** **(%)**	**At index**	***N*** **(%)**
**SSRIs**	**Total**	**367 (29.5)**	**502 (40.4)**	**748 (60.2)**
	Sertraline HCl	155 (12.5)	189 (15.2)	342 (27.5)
	Fluoxetine HCl	114 (9.2)	122 (9.8)	201 (16.2)
	Escitalopram oxalate	83 (6.7)	111 (8.9)	211 (17.0)
	Citalopram hydrobromide	37 (3.0)	59 (4.8)	120 (9.7)
	Paroxetine HCl	27 (2.2)	47 (3.8)	73 (5.9)
	Fluvoxamine maleate	3 (0.2)	4 (0.3)	5 (0.4)
	Paroxetine mesylate	0 (0)	2 (0.2)	2 (0.2)
**SNRIs**	**Total**	**440 (35.4)**	**366 (29.5)**	**535 (43.1)**
	Duloxetine HCl	294 (23.7)	215 (17.3)	334 (26.9)
	Desvenlafaxine succinate	103 (8.3)	57 (4.6)	103 (8.3)
	Venlafaxine HCl	94 (7.6)	103 (8.3)	173 (13.9)
	Levomilnacipran HCl	22 (1.8)	12 (1.0)	27 (2.2)
	Desvenlafaxine	0 (0)	1 (0.1)	3 (0.2)
	Desvenlafaxine fumarate	0 (0)	0 (0)	0 (0)
**Other (NDRIs and tetracyclics)**	**Total**	**420 (33.8)**	**417 (33.6)**	**564 (45.4)**
	Bupropion HCl	419 (33.7)	415 (33.4)	564 (45.4)
	Bupropion hydrobromide	2 (0.2)	2 (0.2)	2 (0.2)
	Maprotiline HCl	0 (0)	0 (0)	0 (0)
Benzodiazepines	NA	246 (19.8)	307 (24.7)	504 (40.6)
**Other (serotonin modulators)**	**Total**	**485 (39.0)**	**298 (24.0)**	**454 (36.6)**
	Trazodone HCl	459 (37.0)	271 (21.8)	410 (33.0)
	Vilazodone HCl	46 (3.7)	40 (3.2)	78 (6.3)
	Nefazodone HCl	2 (0.2)	1 (0.1)	4 (0.3)
Antihyperlipidemics	NA	330 (29.0)	352 (28.3)	412 (33.2)
Antipsychotics	NA	328 (26.4)	191 (15.4)	298 (24.0)
Nonbenzodiazepine hypnotics	NA	276 (22.2)	233 (18.8)	393 (31.6)
Antihypertensives	NA	345 (27.8)	278 (22.4)	351(28.3)
Beta blockers	NA	237 (19.1)	218 (17.6)	266 (21.4)
Other (tetracyclics)	Mirtazapine	147 (11.8)	90 (7.2)	169 (13.6)
Tricyclic agents	NA	192 (15.5)	103 (8.3)	186 (15.0)
Diuretics	NA	164 (13.2)	158 (12.7)	202 (16.3)
Misc. hematological	NA	152 (12.2)	152 (12.2)	178 (14.3)
Antidiabetics	NA	129 (10.4)	127 (10.2)	144 (11.6)
Calcium blockers	NA	109 (8.8)	100 (8.1)	132 (10.6)
Anticoagulants	NA	38 (3.1)	35 (2.8)	60 (4.8)
Antiarrhythmics	NA	17 (1.4)	17 (1.4)	22 (1.8)
Other (MAOI)	Tranylcypromine sulfate	2 (0.2)	1 (0.1)	4 (0.3)
Other (MAOI)	Isocarboxazid	0 (0)	0 (0)	0 (0)
Other (MAOI)	Phenelzine sulfate	0 (0)	0 (0)	0 (0)

*Historical*: Medications that the patient was on the day before the “Index date.” If the patient’s first encounter at Parkview is on the Index date or if the medication is self-reported. *Index*: Medications that the patient was on specifically at the “Index date” encounter. *Concurrent*: Medications that the patient was on any time between 4 weeks after the “Index date” and their “Follow-up date”

NDRI, norepinephrine and dopamine reuptake inhibitor; MAOI, monoamine oxidase inhibitor; NA, not applicable; SNRI, serotonin-norepinephrine reuptake inhibitor; SSRI, selective serotonin reuptake inhibitor

### Depression severity

On average, PHQ-9 scores decreased by 4.39 points from index to 12 weeks (95% CI: 4.03 to 4.76). At 12 weeks, PHQ-9 mean scores decreased significantly from baseline, from 14.15 ± 5.8 to 9.62 ± 6.03; *p* < 0.001; Cohen’s *d* = 0.73 (Table 5).

**Table 5:** Descriptives at index and 12 weeks and mean differences for all outcome variables

	Index			12 Weeks			*t* test			
	*N*	Mean (SD)	Median (min-max)	*N*	Mean (SD)	Median (min-max)	*N*	*t*	*p*	Effect size^a^
**Outcome**										
PHQ-9	1130	14.15 (5.80)	14 (0–27)	1110	9.62 (6.03)	9 (0–27)	1063	23.72	0.000	0.728
GAD-7	729	11.48 (5.59)	12 (0–21)	735	8.3 (5.51)	8 (0–21)	630	13.78	0.000	0.549
Weight	1208	192.04 (51.68)	186.70 (88.60–492.60)	1204	192.99 (51.45)	188.0 (89.0–484.60)	1176	–3.84	0.000	0.112
BMI	1207	30.26 (7.45)	29.28 (15.21–69.67)	1203	30.41 (7.41)	29.41 (15.28–68.54)	1175	–3.86	0.000	0.113
Sexual dysfunction (PRISE)	371	0.72 (0.81)	0 (0–2)	238	0.50 (0.74)	0 (0–2)	163	2.81	0.006	0.220
Sleep disturbance (PRISE)	370	1.25 (0.76)	1 (0–2)	236	0.90 (0.78)	1 (0–2)	162	3.08	0.002	0.242
Cognitive function (PDQ-20)	309	35.48 (17.13)	35 (0–76)	184	27.88 (17.54)	25 (0–73)	101	5.91	0.000	0.588
Work/Social function (WSAS)	279	19.75 (9.72)	21 (0–40)	149	15.08 (10.35)	14 (0–38)	70	2.36	0.021	0.282
**Clinical notes**										
Sexual dysfunction	1242	0.19 (0.38)	0 (0–3)	1233	0.04 (0.26)	0 (0–3)	1233	9.85	0.000	0.280
Appetite	1242	0.19 (0.48)	0 (0–3)	1233	0.04 (0.26)	0 (0–3)	1233	9.85	0.000	0.430
Sleep disturbance	1242	2.21 (1.08)	2 (0–4)	1233	1.46 (0.98)	2 (0–4)	1233	19.87	0.000	0.566
Absenteeism	824	0.10 (0.31)	0 (0–1)	812	0.10 (0.30)	0 (0–1)	807	0.73	0.466	0.026
Presenteeism	824	1.39 (0.84)	1 (0–3)	812	0.81 (0.66)	1 (0–3)	807	17.34	0.000	0.610

^a^Effect size (Cohen’s *d*) is calculated as the mean difference divided by the SD of the difference

BMI, body mass index; GAD-7, Generalized Anxiety Disorder-7; PDQ-20, Perceived Deficits Questionnaire-20; PHQ-9, Patient Health Questionnaire-9; PRISE, Patient-Rated Inventory of Side Effects; WSAS, Work and Social Adjustment Scale

### Response and remission

**Fig. 1:**
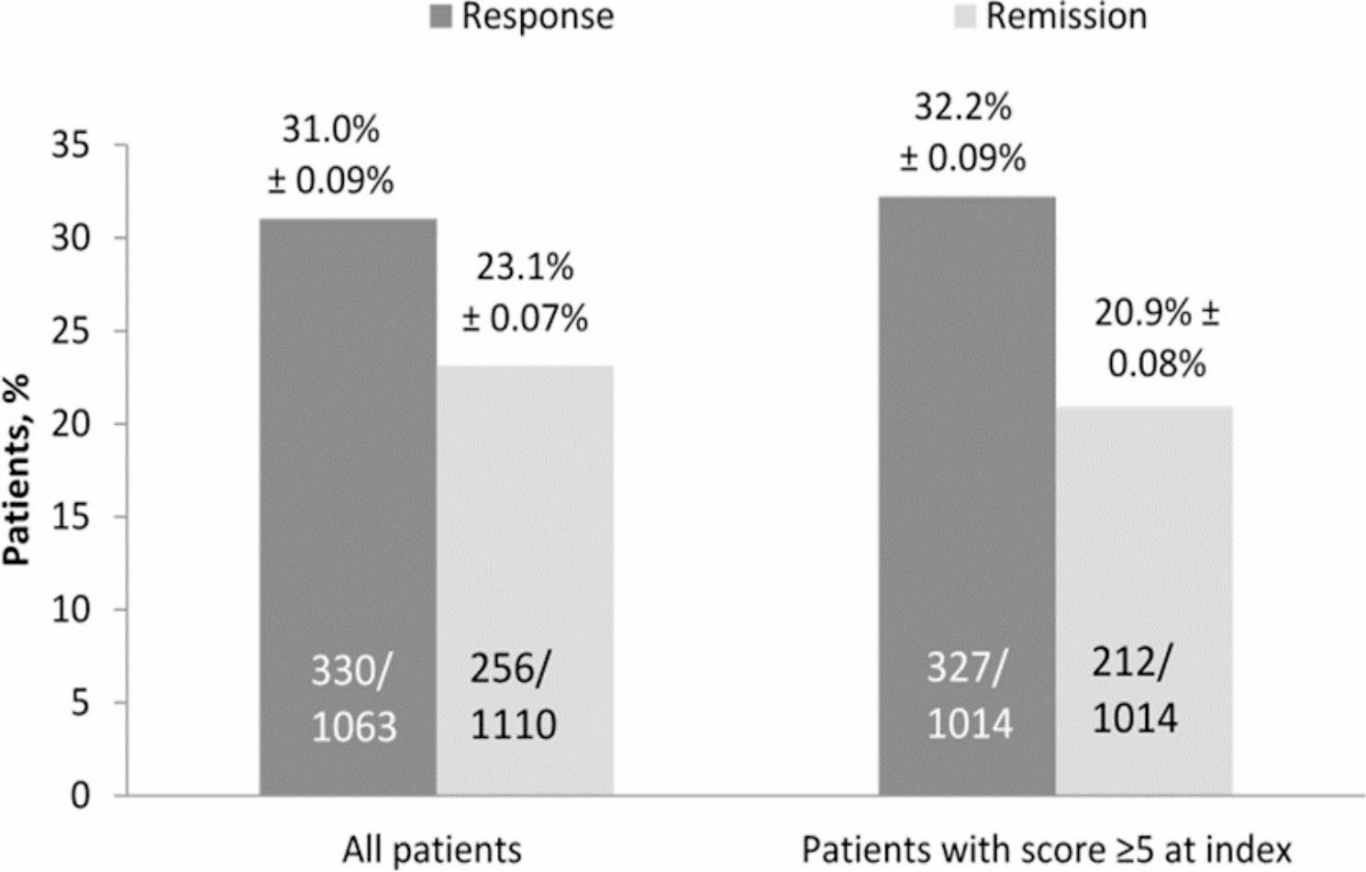
Response and remission rates at 12 weeks based on PHQ-9 scores. *Note*: We also examined patients who began at index with PHQ-9 scores of 5 or greater, as the definition of remission was that patients’ PHQ-9 scores decreased to below 5. Thus, this sensitivity analysis excludes patients who were already meeting the definition of remission at index. PHQ-9, Patient Health Questionnaire-9

At 12 weeks, the response and remission rates in all patients were 31.0% and 23.1%, respectively (Fig. 1). In patients who started at index with a PHQ-9 score ≥ 5, 32% and 21% experienced clinical response and remission at 12 weeks, respectively.

### Anxiety symptoms

On average, GAD-7 scores decreased significantly by 3.13 points (95% CI: 2.69 to 3.58). Mean GAD-7 score changed from 11.48 ± 5.59 to 8.3 ± 5.51; *p* < 0.001, with a medium effect size of Cohen’s *d* = 0.55 (Table 5).

### Perception of cognitive functioning, sleep disturbance, and sexual dysfunction

In a smaller subsample of patients for whom data were available, cognitive symptoms (*n* = 309; Mean_Diff_ = 7.74, 95% CI: 5.14 to 10.34; *p* < 0.001; Cohen’s *d* = 0.59), sleep disturbance (*n* = 370; Mean_Diff_ = 0.25, 95% CI: 0.09 to 0.42; *p* = 0.002; Cohen’s *d* = 0.24), and sexual dysfunction (*n* = 371; Mean_Diff_ = 0.18, 95% CI: 0.05 to 0.30; *p* = 0.006; Cohen’s *d* = 0.22) decreased significantly at 12 weeks (Table 5). Scores for sleep disturbance (Mean_Diff_ = 0.75, 95% CI: 0.67 to 0.82; *p* < 0.001; Cohen’s *d* = 0.57) and sexual dysfunction (Mean_Diff_ = 0.15, 95% CI: 0.12 to 0.18; *p* < 0.001; Cohen’s *d* = 0.28) also showed significant improvements, as revealed by a review of clinical notes in the full sample.

### Change in absenteeism and presenteeism

Clinical notes revealed a significant decrease in symptoms (Mean_Diff_ = 0.59, 95% CI: 0.53 to 0.66; *p* < 0.001; Cohen’s *d* = 0.61) related to presenteeism (for the 826 and 813 patients listed as currently employed or as students at index and at 12 weeks, respectively) (Table 5). No significant change in absenteeism was observed (Mean_Diff_ = 0.01, 95% CI: -0.02 to 0.04; *p* < 0.466; Cohen’s *d* = 0.03).

### Change in work/social functioning

WSAS scores decreased significantly, on average by 2.51 points by 12 weeks (95% CI: 0.39 to 4.64; *p* = 0.021; Cohen’s *d* = 0.28) (Table 5).

### Change in weight/BMI

Weight and BMI showed statistically significant increases of an average of 0.87 pounds (95% CI: 0.42 to 1.31) or 0.14 BMI points (95% CI: 0.07 to 0.21) by 12 weeks (*p* < 0.001; Cohen’s *d* = 0.11).

### Change in appetite

The clinical notes revealed that appetite improved significantly by 12 weeks (Mean_Diff_ = 0.45, 95% CI: 0.39 to 0.51; *p* < 0.001; Cohen’s *d* = 0.43).

### Vortioxetine persistence

At 12 weeks, 66.9% (832/1242) of patients continued on vortioxetine treatment.

## Discussion

In this retrospective real-world study conducted at a single clinical outpatient psychiatric practice site, we reviewed charts from 1242 outpatients with MDD who had initiated vortioxetine and had visit data over approximately 12 weeks. Most patients included in this study had prior antidepressant failure and several psychiatric and medical comorbidities.

At 12 weeks, patients demonstrated statistically and clinically significant improvement in depression severity, anxiety, sleep, work/social functioning (small effect size in a smaller sample size), appetite, and perception of cognitive dysfunction without worsening of sexual function. Weight gain of less than 1 pound per 12 weeks, which was statistically significant, was observed in our sample [6]. Although other published placebo-controlled trials and open‐label extension studies with vortioxetine have not reported a significant effect on body weight in either short- or long-term studies, weight gain has been identified during post-approval use [7].

At 12 weeks, two-thirds of patients continued on vortioxetine treatment. Of note, a recent retrospective analysis in Italy also found that patients may have a lower risk of low adherence when being treated with vortioxetine compared with many other antidepressants [38]. Within our study, about 1 in 3 patients showed clinical response. The remission rate at 12 weeks was 23%, which, although lower than that reported in clinical trials (29–38%) [39], could still be interpreted as encouraging considering that 67% of the study population had recurrent moderate MDD, multiple comorbidities, and previous antidepressant therapy had failed. Moreover, in the STAR*D (Sequenced Treatment Alternatives to Relieve Depression) trial, which also included patients with comorbid diagnoses and used an MBC approach employing the 16-item Quick Inventory of Depressive Symptomatology–Self-Report (QIDS-SR16 16), remission rates (score ≤ 5) were less than 15% in the third and fourth lines of treatment. The disparity in measures of treatment success is also apparent when reviewing results from a recent open-label clinical study evaluating the effectiveness of vortioxetine in patients with moderately severe depression. Using a goal attainment scaling approach as the primary outcome measure, 57.8% of patients achieved their goals by week 12, whereas approximately 40% of patients achieved remission based on standardized clinician-reported Clinical Global Impressions-Severity and patient-reported (PHQ-9) scales. This discrepancy between measures of treatment success suggests the need for a closer look at how these measures reflect a patient’s overall response and functional recovery.

One of the unique features of our study was the use of PROMs to measure meaningful treatment progress not only for depression, but also for anxiety, cognition, and functional outcomes. The use of PRISE and narrative note review provided additional information concerning insomnia and sexual disturbances. The primary outcome, PHQ-9 improvement at 12 weeks, was statistically and clinically significant, indicating meaningful treatment progress. This is especially true considering that this study population of patients with MDD had multiple comorbid psychiatric and physical diagnoses and multiple previous antidepressant treatments that failed. In addition, using the guideline of an effect size > 0.5 [32], representing moderate clinical significance, an effect size of 0.728 was shown for improvement in depression. Effect sizes of 0.2 − 0.5 are considered small, 0.5 − 0.8 moderate, and > 0.8 large in psychopharmacology studies [40].

GAD was the most frequent comorbid psychiatric diagnosis prior to starting vortioxetine treatment, occurring in 82% of patients in our study. Patients with MDD comorbid with GAD make up a patient population that is more difficult to treat than patients with a diagnosis of MDD or GAD alone [41]. Compared with patients with only depression, patients who have depression and comorbid anxiety have greater severity of illness, higher chronicity rates, and significantly greater impairment in quality of life [41].

In our analysis, symptoms of anxiety improved from moderate at baseline to mild at 12 weeks, with a medium effect size (Cohen’s *d* = 0.55) [4–6]. This is similar to the results observed in another real-world study, which reported significant improvement in the severity of anxiety symptoms, from “severe” anxiety at baseline to “mild” over 52 weeks of vortioxetine treatment [8]. Furthermore, in another recent open-label study in adult outpatients with severe MDD and severe comorbid GAD (RECONNECT; ClinicalTrials.gov ID: NCT04220996), clinically meaningful and statistically significant improvements from baseline in symptoms of depression and anxiety and overall functioning and health-related quality of life were observed after 8 weeks of vortioxetine treatment with a starting dose of 10 mg/day and forced up-titration to 20 mg/day after 1 week [42]. In addition, the relatively low dosage (70% in this study received < 20 mg) in a naturalistic study affirms that the efficacy achieved from vortioxetine appears to relate to 5 selective receptor affinities (1 A agonism, 2B and 2D partial agonism, and 3 and 7 antagonism) despite lower 5HT transporter inhibition compared with an SSRI. For instance, SSRIs at therapeutic doses provide 80% reuptake inhibition, whereas vortioxetine provides 65% at 10 mg and 80% at 20 mg. Lower doses in naturalistic settings achieve reasonable efficacy while limiting potential intolerance at higher doses [43–45].

In addition to improvement in anxiety symptoms, other secondary outcomes with moderate effect sizes included improvement of perceived cognitive dysfunction and sleep disturbances or insomnia. Significant improvements in patient-rated cognitive symptoms, work productivity, and functional outcomes were also reported in a Canadian study performed in a real-world setting after continuous long-term vortioxetine treatment for up to 52 weeks [8].

Similar to previous studies demonstrating that switching to vortioxetine improved sexual dysfunction associated with previous SSRI use [46, 47], a mild effect size for sexual dysfunction improvement was seen in our study as well. This improvement in sexual dysfunction occurred despite our study not recruiting patients with prior sexual disturbances. Improvement in workplace productivity was demonstrated by improvements in levels of presenteeism, but not absenteeism, after 12 weeks of vortioxetine treatment. Overall, our study results thus suggested improvements in depression severity, anxiety, sleep, work/social functioning, appetite, sexual dysfunction, and perception of cognitive dysfunction—which might be due to the effectiveness of being treated with vortioxetine (although it may be that other factors were also involved in these outcome changes, as this study was not a controlled trial). In addition, our study demonstrated the utility and practicality of PROM metrics and MBC to monitor meaningful treatment progress.

A high proportion of patients in our study had multiple comorbidities at baseline, which would have led to their exclusion from the majority of clinical trials. About 64% had 3 or more psychiatric diagnoses, and about 25% had 3 or more other comorbidities. The high prevalence of psychiatric comorbidities reported in our study is also consistent with previous reports of association of various psychiatric comorbidities with MDD [1]. The presence of psychiatric comorbidities in MDD is associated with greater disease severity, recurrence, poor functioning, and suicidality [1]. In addition, our study population was characterized by recurrent moderate MDD; at study onset, about 60% of patients had a prescription for SSRIs, 43% were using SNRIs, and 45% were using NDRIs and tetracyclics. Long duration of MDD together with psychiatric and medical comorbidities are the main contributors to treatment-resistant depression and are associated with higher mortality [48]. Thus, the improvements in depression and anxiety seen in our study are likely clinically meaningful, especially considering the clinical complexity of the study population in this analysis.

Despite the demonstrated benefits of MBC in improving treatment outcomes and patient engagement, adoption of MBC in routine clinical practice has been slow [49]. In a survey of 314 psychiatrists, some of the reasons for not following MBC included taking too much time (34%), not being trained to use them (34%), not believing it would be clinically helpful (21%), not knowing which scales to use (21%), and being too disruptive of clinical practice (19%) [50]. In this regard, PROMs may be able to address many of the perceived and actual barriers to implementing MBC because of their efficiency and ease of use [49]. Several professional societies have established guidance for the collection and reporting of PROMs, including the National Quality Forum and the National Institutes of Health, which funded the Patient Reported Outcomes Measurement Information System [51, 52]. Integration of PROMs into care pathways and electronic health records (EHRs), when possible, can achieve more standardized and efficient clinical documentation and workflow, as well as improving communication between providers and patients [49]. Many healthcare providers now have access to EHRs, some of which have incorporated measurements for mental health outcomes with dashboards that allow tracking of scores over time [49]. Recently, use of an MBC program called VitalSign6, which enabled integration with EHRs, showed a statistically significant decrease in self-reported depression scores from baseline to follow-up and was effective in improving identification and management of depression in primary care [53, 54].

### Study limitations

Although the overall results of this study suggest potentially positive effects of vortioxetine treatment, in the absence of a comparison group of patients with MDD who began other medications for the treatment of depression, we cannot attribute the observed improvement to vortioxetine alone. Additionally, the current patient sample is primarily White (91.1%), and results may not be generalizable to other races and ethnicities. Although we still had enough statistical power to detect significant effects, the sample size was much smaller for some of the scales (i.e., PRISE, PDQ-20, WSAS compared with PHQ-9 and GAD-7), and it is possible that the effect sizes may be a bit inflated due to the possible attrition of some patients in the analyses (e.g., perhaps those for whom we did not have a visit at the 12-week follow-up were those whose symptoms were deteriorating). Additionally, even though the focus of the current analysis was on change by 12 weeks, some patients may have had one to several encounters between the initial encounter and the encounter closest to 12 weeks. An improvement or deterioration toward the domains, especially in the clinical narrative notes, might have been noted in one of these in-between encounters but not reiterated in the encounter closest to 12 weeks. Moreover, although it is likely that those with a prescription order for vortioxetine indeed began taking the medication, without information on medication compliance it is possible that some patients within our sample never began taking vortioxetine or did not comply with taking vortioxetine; our current data suggests that about 33% of patients no longer had a vortioxetine prescription by the 12-week follow-up. Yet, we treated our analyses as intention-to-treat analyses where all patients who had received a prescription of vortioxetine were included in the analyses regardless of whether they complied or persisted in their use of vortioxetine.

The analysis of the clinical notes presented limitations as well. While we were able to overcome many of the inconsistencies commonly observed when using health system data across thousands of patients, it is possible that inconsistencies between clinical narrative notes and outcome scales could produce measurement error at times in the algorithm and narrative data. As such, inferential statistics run on the scores generated by our algorithm might include additional error not seen in well-validated scales. Nevertheless, we are confident that much of our algorithm for examining the clinical notes performed satisfactorily; further refinement of the keywords and computing methods used could also improve the performance of the algorithm. In the future, it would be important to increase the validation sample to at least 200 encounters, as this would provide greater confidence in the possible validity of the scoring, especially in categories that were not frequently present in our current validation subsample.

## Conclusions

Results suggest that patients with MDD who had multiple comorbid psychiatric and physical diagnoses and multiple previous antidepressant treatment failures showed significant improvements in symptoms of depression and anxiety following 12 weeks of vortioxetine treatment. In addition, patients treated with vortioxetine showed significant improvements in sleep disturbances, sexual dysfunction, and perceptions of cognitive dysfunction. At 12 weeks, in these patients with complex MDD, about 1 in 3 patients showed clinical response, and two-thirds of patients continued vortioxetine treatment. In addition, our study demonstrated the utility and practicality of PROMs and MBC to monitor meaningful treatment progress.

### Electronic supplementary material

Below is the link to the electronic supplementary material.

Additional file 1

## Data Availability

The datasets generated during and/or analyzed during the current study are not publicly available due to Parkview Health’s confidentiality policy. As approved by the Parkview Health Institutional Review Board, the data for this study are only accessible to the approved study team and only aggregated study results can be shared. Requests for aggregated study results can be sent to Jay Fawver at Jay.Fawver@Parkview.com.

## Footnotes

1 A prescription of vortioxetine was defined as a patient having a prescription order and dosage listed in their medical record.

## Abbreviations

BMI: body mass index
EHRs: electronic health records
GAD: generalized anxiety disorder
GAD-7: Generalized Anxiety Disorder-7
HEDIS: Healthcare Effectiveness Data and Information Set
HSIR: Health Services and Informatics Research team
MBC: measurement-based care
MDD: major depressive disorder
NCQA: The National Committee for Quality Assurance
NDRIs: norepinephrine and dopamine reuptake inhibitors
PDQ-20: Perceived Deficits Questionnaire-20
PHQ-9: Patient Health Questionnaire-9
PRISE: Patient-Rated Inventory of Side Effects
PROMIS: Patient Reported Outcomes Measurement Information System
PROMs: patient-reported outcome measures
QIDS-SR16: 16-item Quick Inventory of Depressive Symptomatology–Self-Report
SOAP: subjective,objective,assessment,plan
SNRIs: serotonin norepinephrine-reuptake inhibitors
SSRIs: selective serotonin reuptake inhibitors
STAR*D: Sequenced Treatment Alternatives to Relieve Depression
WSAS: Work and Social Adjustment Scale
