# Supplementary material for: Real-world clinical outcomes and treatment patterns in patients with MDD treated with vortioxetine: a retrospective study
Source: BMC Psychiatry. 2023 Dec 13;23:938. doi: 10.1186/s12888-023-05439-8 (PMC10720213; doi:10.1186/s12888-023-05439-8)
Supplement: Supplementary file 1 — Additional file 1 [file 12888_2023_5439_MOESM1_ESM.docx]

**Additional material for:**

**Real-world clinical outcomes and treatment patterns in patients with MDD treated with vortioxetine:
A retrospective study**

McDaniel BT, et al.

**Additional Table 1.** Validation Results for the Overall Scores by Domain

| Domain | n encounters reviewed | Mean actual  score | Mean predicted score | Median actual  score | Median predicted score | % Correct |
| --- | --- | --- | --- | --- | --- | --- |
| Absenteeism | 50 | 0.08 | 0.06 | 0 | 0 | 98.0 |
| Appetite | 50 | 0.72 | 0.68 | 0 | 0 | 96.0 |
| Presenteeism | 50 | 1.22 | 1.04 | 1 | 1 | 82.0 |
| Sleep | 50 | 1.76 | 1.80 | 2 | 2 | 84.0 |
| Sexual dysfunction | 100 | 0.38 | 0.37 | 0 | 0 | 97.0 |

**Additional Table 2****.** Validation Results for Individual Category Scores Under Various Domains

| Category | n records reviewed | True positives | True negatives | False positives | False negatives | Error count | Error rate | Sensitivity | Specificity | Precision | Accuracy |
| --- | --- | --- | --- | --- | --- | --- | --- | --- | --- | --- | --- |
| Sexual Dysfunction Domain | | | | | | | | | | | |
| Arousal | 100 | 1 | 99 | 0 | 0 | 0 | 0.00 | 1.00 | 1.00 | 1.00 | 1.00 |
| Desire | 100 | 24 | 75 | 0 | 1 | 1 | 0.01 | 0.96 | 1.00 | 1.00 | 0.99 |
| General functioning | 100 | 11 | 87 | 1 | 1 | 2 | 0.02 | 0.92 | 0.99 | 0.92 | 0.98 |
| Orgasm^a^ | 100 | 0 | 100 | 0 | 0 | 0 | 0.00 | − | 1.00 |  | 1.00 |
| Appetite Domain | | | | | | | | | | | |
| Craving/Lack of control | 50 | 7 | 42 | 0 | 1 | 1 | 0.02 | 0.88 | 1.00 | 1.00 | 0.98 |
| Decreased appetite | 50 | 8 | 40 | 1 | 1 | 2 | 0.04 | 0.89 | 0.98 | 0.89 | 0.96 |
| Increased appetite | 50 | 6 | 44 | 0 | 0 | 0 | 0.00 | 1.00 | 1.00 | 1.00 | 1.00 |
| Other | 50 | 13 | 37 | 0 | 0 | 0 | 0.00 | 1.00 | 1.00 | 1.00 | 1.00 |
| Satiety | 50 | 0 | 50 | 0 | 0 | 0 | 0.00 |  | 1.00 |  | 1.00 |
| Variable appetite | 50 | 1 | 49 | 0 | 0 | 0 | 0.00 | 1.00 | 1.00 | 1.00 | 1.00 |
| Change in appetite (combination of decreased/increased/ variable appetite) | 50 | 14 | 35 | 0 | 1 | 1 | 0.02 | 0.93 | 1.00 | 1.00 | 0.98 |
| Absenteeism Domain | | | | | | | | | | | |
| Absenteeism | 50 | 3 | 46 | 0 | 1 | 1 | 0.02 | 0.75 | 1.00 | 1.00 | 0.98 |
| Presenteeism Domain | | | | | | | | | | | |
| Complete tasks, mistakes, organization | 50 | 2 | 45 | 0 | 3 | 3 | 0.06 | 0.40 | 1.00 | 1.00 | 0.94 |
| Focus/Concentration | 50 | 31 | 14 | 0 | 5 | 5 | 0.10 | 0.86 | 1.00 | 1.00 | 0.90 |
| Other symptoms | 50 | 18 | 29 | 1 | 2 | 3 | 0.06 | 0.90 | 0.97 | 0.95 | 0.94 |
| Sleep Category Domain | | | | | | | | | | | |
| Amount of sleep | 50 | 11 | 39 | 0 | 0 | 0 | 0.00 | 1.00 | 1.00 | 1.00 | 1.00 |
| Daytime sleepiness | 50 | 31 | 14 | 3 | 2 | 5 | 0.10 | 0.94 | 0.82 | 0.91 | 0.90 |
| Sleep disturbance | 50 | 32 | 17 | 1 | 0 | 1 | 0.02 | 1.00 | 0.94 | 0.97 | 0.98 |
| Time to fall asleep | 50 | 10 | 36 | 2 | 2 | 4 | 0.08 | 0.83 | 0.95 | 0.83 | 0.92 |

^a^Sensitivity and precision cannot be calculated for orgasm because it was never encountered in the reviewed sample.
